# Sociodemographic, behavioral, and obstetric factors associated with preterm birth and its severity: A matched case-control study in Cyprus

**DOI:** 10.18332/ejm/224190

**Published:** 2026-07-24

**Authors:** Lenos Mastrou, Maria Kyprianidou, Demetris Lamnisos, Konstantinos Giannakou

**Affiliations:** 1 Department of Health Sciences, School of Sciences, European University Cyprus, Nicosia, Cyprus

**Keywords:** preterm birth, maternal risk factors, obstetric history, sociodemographic determinants, Cyprus

## Abstract

**INTRODUCTION:**

Preterm birth (PTB), defined as delivery before 37 weeks of gestation, is a major cause of neonatal mortality and morbidity worldwide. Its multifactorial etiology remains incompletely understood, and Cyprus reports among the highest PTB rates in Europe, highlighting the need for population-specific evidence. This study aimed to investigate the sociodemographic, behavioral, and obstetric history factors of PTB in Cyprus and to examine whether these factors differed according to the degree of prematurity.

**METHODS:**

A matched case-control study was conducted at a tertiary referral hospital in Cyprus, designated as the national center for maternal and neonatal care. Eligible participants were women delivering between January 2019 and December 2022. Conditional logistic regression was conducted to determine the significant associations between sociodemographic, behavioral and obstetric history with PTB. Subgroup analysis of extreme to very (<32 weeks) vs moderate to late PTB (32 to <37 weeks) was also conducted.

**RESULTS:**

Participants were 978 women (489 cases <37 weeks, 489 controls ≥37 weeks). Maternal age increased PTB odds (AOR=1.21; 95% CI: 1.06–1.38), as did previous gynecological surgeries (AOR=1.74; 95% CI: 1.30–2.34), cesarean section (AOR=1.87; 95% CI: 1.28–2.73), and miscarriage (AOR=1.44; 95% CI: 1.03–2.00). Primiparous women had reduced odds versus nulliparous (AOR=0.71; 95% CI: 0.52–0.98). In subgroup analysis, higher BMI (AOR=0.96; 95% CI: 0.92–0.99), prior cesarean (AOR=0.54; 95% CI: 0.30–0.99), and asylum seeker status (AOR=0.19; 95% CI: 0.05–0.67) were linked to lower odds of extreme PTB.

**CONCLUSIONS:**

Future prospective studies are needed to further investigate and confirm the observed associations between sociodemographic, behavioral, and obstetric factors with PTB and degree of prematurity.

## INTRODUCTION

According to the World Health Organization (WHO), preterm birth (PTB) is defined as delivery before 37 completed weeks of gestation or before 259 days from the last menstrual period. PTBs are categorized as extremely preterm (<28 weeks), very preterm (28 to <32 weeks), and moderate to late preterm (32 to <37 weeks)^1^. Globally, an estimated 13.4 million newborns (9.9% of all live births) were born preterm in 2020, with little change over the past decade^2^. PTB remains a persistent challenge, representing both an adverse pregnancy outcome and, in some cases, a medically necessary intervention to prevent severe maternal or neonatal complications^3^.

Multiple risk factors contribute to PTB. Non-modifiable factors include prior PTB, advanced maternal age, multiple pregnancies, and cervical insufficiency. Modifiable determinants such as maternal nutrition, socioeconomic status, obesity, lifestyle behaviors, intrauterine infection, and access to prenatal care are crucial targets for prevention^4^. Despite extensive research, the pathophysiology of PTB is incompletely understood and likely reflects both activation of normal labor pathways and diverse pathological insults^5^. PTB is the leading cause of neonatal mortality and the second leading cause of death in children aged <5 years: accounting for 18% for children aged <5 years and 35% of neonatal deaths^5^. Survivors are at increased risk of long-term complications, including neurodevelopmental disability, learning difficulties, and emotional or social immaturity^6,7^. Beyond the human burden, PTB imposes substantial economic costs globally through medical care, special education, and reduced productivity^1^.

Although PTB is a global concern, Cyprus reports disproportionately high rates in Europe, reaching 13.3% in 2023^6^, a markedly higher rate compared with the median 6.8% in Europe^8^. Limited research in Cyprus has identified advanced maternal age, emotional stress, and long working hours as risk factors for PTB^7^. Most existing studies, however, have focused primarily on neonatal complications rather than maternal or contextual determinants. The persistently high incidence of PTB underscores the urgent need to investigate sociodemographic, lifestyle, and obstetric factors specific to the Cypriot population. Addressing this gap is crucial for informing public health strategies and guiding targeted interventions to reduce PTB prevalence in Cyprus.

Therefore, the present study aimed to investigate the sociodemographic, behavioral, and obstetric history factors of PTB in Cyprus, where evidence remains limited despite persistently high PTB rates. The study also examined whether these factors differed between extreme to very preterm births and moderate to late preterm births, to provide context-specific evidence and improve understanding of PTB determinants in similar populations.

## METHODS

### Study design and setting

This matched case-control study, based on hospital medical records, was conducted at a tertiary referral hospital in Nicosia, Cyprus, designated as the national center for maternal and neonatal care under the State Health Services Organization (SHSO). It is the country’s only public hospital dedicated to mother-and-child health and the sole tertiary referral unit for neonatal intensive care. The Neonatal Intensive Care Unit (NICU) accepts referrals from all public and private maternity facilities across Cyprus, thereby managing most of the island’s high-risk pregnancies and neonatal cases. This centralized referral function ensures that the study population is representative of the national burden of complex neonatal care. The study followed the STROBE (Strengthening the Reporting of Observational Studies in Epidemiology) guidelines^9^ (**Supplementary file**).

### Participants and matching

**Figure 1:**
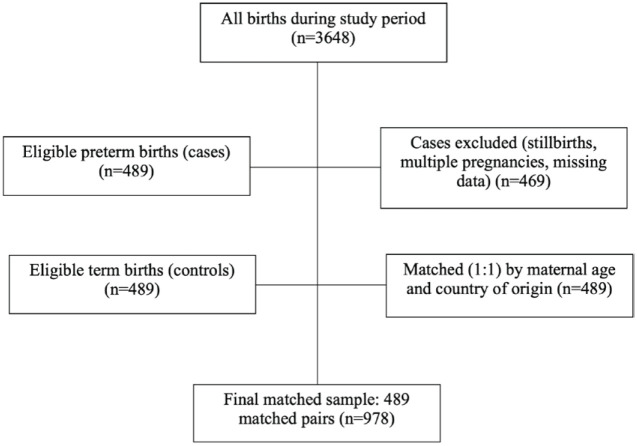
Flowchart of participant selection and matching procedure for the matched case–control study of preterm birth in Cyprus, 2019–2022 (N=978)

Eligible participants were women who gave birth at the tertiary referral hospital between January 2019 and December 2022. Exclusion criteria included stillbirths, multiple pregnancies, or incomplete medical records (Figure 1). Cases of PTB were identified through the hospital’s annual birth registry, and corresponding medical records were retrieved using each patient’s unique identification number. Controls (term births) were selected using a 1:1 matching ratio. Matching was performed on maternal age (±3 years) and country of origin because both factors are known to be associated with preterm birth risk^2,10^ and may act as important confounding variables within the Cypriot population. This approach aimed to reduce potential confounding and improve comparability between cases and controls.

### Data sources

Records missing essential information across key domains – sociodemographic, lifestyle, or obstetric history – were excluded. However, initially missing files were documented for possible inclusion upon re-identification. The study population comprised women who delivered singleton preterm infants at the tertiary hospital between January 2019 and December 2022.

### Ethics

Ethical approval was obtained from the Cyprus National Bioethics Committee (CNBC) (Approval number: EEBK EΠ 2022.01.300; Date: December 2022) and from the Research and Innovation Centre of SHSO (Approval number: 2/23; Date: February 2023). The application, describing the study objectives, data collection and management procedures, intended data use, and expected benefits, was submitted to CNBC and SHSO. All patient data were anonymized before analysis, with unique codes replacing identifiable information to maintain confidentiality.

### Variables and definitions

PTB (case group) was defined as live birth before 37 completed weeks of gestation, while term birth (control group) was defined as live birth at or beyond 37 completed weeks. PTB cases were further categorized into two subgroups: 1) extreme to very preterm (<32 weeks); and 2) moderate to late preterm (32 to <37 weeks)^1^. Gestational age at delivery was obtained from medical records, as documented by healthcare professionals using standard obstetric dating methods. Missing data were handled using available-case analysis, and the number of observations available for each variable is reported within the corresponding tables.

*Potential covariates*

Potential covariates were grouped into three domains: sociodemographics (maternal age, country of origin, education level, marital status, paternal country); health behaviors (maternal weight, height, BMI, smoking – during or before pregnancy, alcohol or drug use – during or before pregnancy); and obstetric/gynecological history (previous cesarean section, abortion, miscarriage), gynecological surgery (cesarean section, abortion procedures, and dilation and curettage – D&C), breastfeeding in previous births, and history of PTB. BMI (kg/m^2^) was calculated based on weight reported in the first prenatal visit. Parity was categorized into nulliparity, primiparity, multiparity, and great multiparity. Maternal age, weight, height, and BMI were analyzed as continuous variables. Smoking, alcohol/drug use, previous cesarean section, miscarriage, abortion, gynecological surgery, breastfeeding history, and history of PTB, were coded as binary variables (yes, no). Maternal age categories, education level, marital status, parity, and country of origin, were analyzed as categorical variables. Data were coded using binary or ordinal scales and entered a secure encrypted Microsoft Excel database provided by the SHSO Research and Innovation Centre, with access restricted to the research team.

### Statistical analysis

Normality of continuous variables was assessed using the Shapiro-Wilk test. Based on the distributional characteristics, continuous variables are summarized as mean with standard deviation (SD) when normally distributed, and median with interquartile range (IQR) when not normally distributed. For the primary comparison (preterm vs term births), continuous variables were analyzed using the Wilcoxon signed-rank test, and binary variables using McNemar’s test. Categorical variables with more than two categories were analyzed using conditional logistic regression, which accounts for the matched design. Covariate balance between cases and controls after matching was assessed using standardized mean difference (SMD), with values <0.1 indicating acceptable balance. For the secondary comparison (extreme to very preterm vs moderate to late preterm), which was unmatched, continuous variables were analyzed using the Mann–Whitney U test, and categorical variables using chi-squared or Fisher’s exact tests. Univariable analyses employed conditional logistic regression for matched data and binary logistic regression for unmatched data. Unadjusted odds ratios (ORs), 95% confidence intervals (CIs), and p-values are reported. Maternal weight, maternal height, and BMI were initially explored as separate variables in univariable analyses; however, because BMI is derived from weight and height, these variables were not simultaneously included within the same adjusted multivariable model.

Separate multivariable models were constructed for the primary matched PTB analysis and the secondary subgroup analysis according to degree of prematurity. Models included variables of theoretical relevance (maternal age, maternal country of origin, education level, parity, smoking, and BMI). Adjusted odds ratios (AORs) and 95% CI are reported. Multicollinearity was assessed using the variance inflation factor (VIF) and tolerance (1/VIF); variables with VIF >2.5 and tolerance <0.2 were excluded. Potential interactions were tested using binary logistic regression. Model calibration was assessed with the Hosmer–Lemeshow test for binary logistic models, while explanatory power was evaluated with pseudo-R² statistics (Cox & Snell, McFadden). Model parsimony was evaluated using the log-likelihood and Akaike information criterion (AIC). Influential observations were identified using Cook’s distance in binary logistic models and leave-one-stratum-out diagnostics in conditional logistic models. All tests were two-sided, and p<0.05 was considered statistically significant. Statistical analyses were performed using IBM SPSS Statistics version 30.0 and R version 4.5.1.

## RESULTS

### Sociodemographic characteristics

A total of 978 women participated, including 489 with PTB and 489 matched controls. Table 1 summarizes the sociodemographic characteristics. The median maternal age was 30.0 years (IQR: 26.0–34.0), with SMD <0.1. More than half of the women (52.4%) were of Cypriot origin, with no variation between groups (p=1.000), as it was an exact matching variable. Most participants (57.7%) had tertiary education, with similar proportions among cases (57.6%) and controls (57.9%) (p=0.779). Employment rates were also comparable (57.0% vs 55.9%; p=0.893). Marital status and paternal origin showed no significant variation between cases and controls.

**Table 1:** Sociodemographic characteristics of women included in a matched case–control study of preterm birth, overall and according to prematurity status and degree of prematurity, in a tertiary referral hospital, Cyprus, 2019–2022 (N=978)

*Characteristics*	*Overall (N=978)* *n (%)*	*Prematurity status (N=978)*			*Degree of prematurity (N=489)*		
		*Preterm birth (<37* *weeks) (N=489)* *n (%)*	*Term birth (≥37 weeks)* *(N=489)* *n (%)*	*p*	*Extreme to very preterm (<32 weeks) (N=142) n (%)*	*Moderate to late* *preterm (32 to <37* *weeks) (N=347)* *n (%)*	*p*
**Maternal age** (years), median (IQR)							
**Maternal age** (years)	30.00 (26.00–34.00)	30.00 (26.00–34.00)	30.00 (26.00–34.00)	<0.1^a^	30.50 (26.00–34.00)	30.00 (26.00–34.00)	0.213^b^
<20	35 (3.6)	18 (3.7)	17 (3.5)	0.506^c^	4 (2.8)	14 (4.0)	0.484^d^
20–34	734 (75.0)	369 (75.5)	365 (74.6)		104 (73.3)	265 (76.4)	
≥35	209 (21.4)	102 (20.8)	107 (21.9)		34 (23.9)	68 (19.6)	
**Maternal education** (N=949)							
Uneducated/primary	56 (5.9)	31 (6.6)	25 (5.2)	0.779^c^	6 (4.4)	25 (7.4)	**0.039^d^**
Secondary	345 (36.4)	169 (35.8)	176 (36.9)		39 (28.9)	130 (38.6)	
Tertiary	548 (57.7)	272 (57.6)	276 (57.9)		90 (66.7)	182 (54.0)	
**Maternal occupation** (N=944)							
Employed	533 (56.5)	269 (57.0)	264 (55.9)	0.893^c^	91 (65.5)	178 (53.5)	**0.002^d^**
Unemployed	161 (17.1)	75 (15.9)	86 (18.2)		16 (11.5)	59 (17.7)	
Housewife	163 (17.3)	82 (17.4)	81 (17.2)		28 (20.1)	54 (16.2)	
Asylum seeker	87 (9.2)	46 (9.7)	41 (8.7)		4 (2.9)	42 (12.6)	
**Marital status** (N=965)							
Married	645 (66.8)	333 (68.9)	312 (64.7)	0.356^c^	104 (74.8)	229 (66.6)	0.053^d^
Engaged/in a relationship	168 (17.4)	79 (16.4)	89 (18.5)		23 (16.5)	56 (16.3)	
Single/divorced	152 (15.8)	71 (14.7)	81 (16.8)		12 (8.6)	59 (17.2)	
**Maternal country**							
Cyprus	512 (52.4)	256 (52.4)	256 (52.4)	1.00^c^	84 (59.2)	172 (49.6)	0.162^d^
Syria	72 (7.4)	36 (7.4)	36 (7.4)		8 (5.6)	28 (8.1)	
Congo	60 (6.1)	30 (6.1)	30 (6.1)		2 (1.4)	28 (8.1)	
India	46 (4.7)	23 (4.7)	23 (4.7)		7 (4.9)	16 (4.6)	
Romania	44 (4.5)	22 (4.5)	22 (4.5)		5 (3.5)	17 (4.9)	
Cameroon	40 (4.1)	20 (4.1)	20 (4.1)		5 (3.5)	15 (4.3)	
Bulgaria	34 (3.5)	17 (3.5)	17 (3.5)		6 (4.2)	11 (3.2)	
Greece	30 (3.1)	15 (3.1)	15 (3.1)		3 (2.1)	12 (3.5)	
Russia	16 (1.6)	8 (1.6)	8 (1.6)		3 (2.1)	5 (1.4)	
Georgia	16 (1.6)	8 (1.6)	8 (1.6)		5 (3.5)	3 (0.9)	
Nigeria	12 (1.2)	6 (1.2)	6 (1.2)		2 (1.4)	4 (1.2)	
Philippines	12 (1.2)	6 (1.2)	6 (1.2)		1 (0.7)	5 (1.4)	
Other *	84 (8.6)	42 (8.6)	42 (8.6)		11(7.7)	31 (8.9)	
**Maternal country**							
Cyprus	512 (52.4)	256 (52.4)	256 (52.4)	1.00^e^	84 (59.2)	172 (49.6)	0.054^d^
Other	466 (47.6)	233 (47.6)	233 (47.6)		58 (40.8)	175 (50.4)	
**Paternal country**							
Cyprus	496 (50.7)	249 (50.9)	247 (50.5)	0.574^c^	84 (59.2)	165 (47.6)	0.064^d^
Syria	85 (8.7)	41 (8.4)	44 (9.0)		9 (6.3)	32 (9.2)	
Unknown father	61 (6.2)	30 (6.1)	31 (6.3)		3 (2.1)	27 (7.8)	
Greece	52 (5.3)	25 (5.1)	27 (5.5)		6 (4.2)	19 (5.5)	
Romania	53 (5.4)	18 (3.7)	35 (7.2)		4 (2.8)	14 (4.0)	
India	33 (3.4)	19 (3.9)	14 (2.9)		6 (4.2)	13 (3.7)	
Cameroon	28 (2.9)	15 (3.1)	13 (2.7)		4 (2.8)	11 (3.2)	
Congo	26 (2.7)	16 (3.3)	10 (2.0)		2 (1.4)	14 (4.0)	
Bulgaria	24 (2.5)	11 (2.2)	13 (2.7)		4 (2.8)	7 (2.0)	
Pakistan	17 (1.7)	9 (1.8)	8 (1.6)		3 (2.1)	6 (1.7)	
Georgia	13 (1.3)	7 (1.4)	6 (1.2)		5 (3.5)	2 (0.6)	
Nigeria	11 (1.1)	7 (1.4)	4 (0.8)		3 (2.1)	4 (1.2)	
Other **	79 (8.1)	42 (8.6)	37 (7.6)		9 (6.3)	33 (9.5)	
**Paternal country**							
Cyprus	496 (50.7)	249 (50.9)	247 (50.5)	0.912^e^	84 (59.2)	165 (47.6)	**0.020^d^**
Other	482 (49.3)	240 (49.1)	242 (49.5)		58 (40.8)	182 (52.4)	

IQR: interquartile range. Percentages are calculated within each category of the corresponding variable. Bold values indicate statistical significance.

a SMD was calculated as the standardized mean difference between cases and controls; values <0.1 indicate good balance.

b Differences between categories of preterm birth (extreme to very preterm vs moderate to late preterm) were analyzed using Mann–Whitney U test.

c Differences between cases (preterm birth) and controls (term birth) were analyzed using conditional logistic regression.

d Differences between categories of preterm birth (extreme to very preterm vs moderate to late preterm) were analyzed using chi-squared test.

e Differences between cases (preterm birth) and controls (term birth) were analyzed using McNemar’s test for paired nominal data. *Includes Nepal, Pakistan, Sri Lanka, Ukraine, Poland, Bangladesh, Egypt, England, Jordan, Somalia, America, Germany, Hungary, Iraq, Iran, Lebanon,

Liberia, Moldova, Palestine, Slovakia, Turkey, and Vietnam. **Includes Egypt, England, Lebanon, Nepal, Sri Lanka, Bangladesh, France, Somalia, Jordan, Liberia, Poland, Russia, Turkey, Philippines, Albania, Iran, Iraq, Kurdistan, Ukraine, Africa, Argentina, Armenia, Belgium, Germany, Guinea, Italy, Morocco, Spain, and Vietnam.

In subgroup analyses, extreme to very PTBs were more frequently associated with Cypriot maternal origin (59.2% vs 49.6%; p=0.054), marriage (74.8% vs 66.6%; p=0.053), employment (65.5% vs 53.5%; p=0.002), and tertiary education (66.7% vs 54.0%; p=0.039). Similarly, paternal Cypriot origin was more common in extreme to very PTBs compared with moderate to late PTBs (59.2% vs 47.6%; p=0.020).

### Health behaviors

Table 2 presents maternal health behaviors. Median maternal weight and height were slightly lower in cases compared with controls (68.0 vs 69.0 kg and 162.0 vs 163.0 cm, respectively), but the differences were not statistically significant (p=0.783 and p=0.165). Median BMI was marginally higher among cases (26.04 vs 25.56 kg/ m²; p=0.258), although this association was not statistically significant. Smoking was reported by 17.0% of cases and 18.6% of controls (p=0.542), while drug or alcohol use during pregnancy was rare in both groups (<1%; p=0.687), with no statistically significant associations observed. When stratified by degree of prematurity, weight, height, and smoking rates were similar across subcategories. BMI showed a non-statistical significance with degree of prematurity (p=0.085).

**Table 2:** Maternal health behaviors and anthropometric characteristics among women participating in a matched case–control study of preterm birth, overall and according to prematurity status and degree of prematurity, in a tertiary referral hospital, Cyprus, 2019–2022 (N=978)

*Characteristics*	*Overall (N=978)* *Median (IQR)*	*Prematurity status (N=978)*			*Degree of prematurity (N=489)*		
		*Preterm birth* *(<37 weeks)* *(N=489)* *Median (IQR)*	*Term birth* *(≥37 weeks)* *(N=489)* *Median (IQR)*	*p*	*Extreme to very* *preterm (<32 weeks)* *(N=142)* *Median (IQR)*	*Moderate to late* *preterm (32 to <37 weeks)* *(N=347)* *Median (IQR)*	*p*
**Maternal weight** (kg)	68.50 (59.00–78.50)	68.00 (60.00–78.10)	69.00 (58.00–79.00)	0.783^a^	68.00 (58.00–76.00)	68.90 (60.00–79.40)	0.216^b^
**Maternal height** (cm)	163.00 (158.00–167.00)	162.00 (158.00–165.50)	163.00 (158.00–167.00)	0.165^a^	162.00 (159.00–165.00)	162.00 (158.00–166.00)	0.557^b^
**Maternal BMI** (kg/m^2^)	25.86 (22.56–29.63)	26.04 (22.79–30.20)	25.56 (22.42–29.39)	0.258^a^	25.53 (22.66–28.30)	26.23 (22.86–30.85)	0.085^b^
**Smoking**, n (%)							
Yes	174 (17.8)	83 (17.0)	91 (18.6)	0.542^c^	24 (16.9)	59 (17.0)	0.978^d^
No	804 (82.2)	406 (83.0)	398 (81.4)		118 (83.1)	288 (83.0)	
**Drugs of abuse/alcohol consumption,** n (%)							
Yes	6 (0.6)	4 (0.8)	2 (0.4)	0.687^c^	1 (0.7)	3 (0.9)	1.000^e^
No	972 (99.4)	485 (99.2)	487 (99.6)		141 (99.3)	344 (99.1)	

IQR: interquartile range. Percentages are calculated within each category of the corresponding variable. BMI: body mass index.

a Differences between cases (preterm birth) and controls (term birth) were analyzed using Wilcoxon Signed-Rank test.

b Differences between categories of preterm birth (extreme to very preterm vs moderate to late preterm) were analyzed using Mann–Whitney U test.

c Differences between cases (preterm birth) and controls (term birth) were analyzed using McNemar’s test for paired nominal data.

d Differences between categories of preterm birth (extreme to very preterm vs moderate to late preterm) were analyzed using chi-squared test.

e Differences between categories of preterm birth (extreme to very preterm vs moderate to late preterm) were analyzed using Fisher’s exact test.

### Obstetric and gynecological history

As shown in Table 3, women with PTB reported higher rates of gynecological surgery (47.9% vs 37.6%; p=0.001) and previous cesarean section (23.9% vs 18.6%; p=0.045) compared to controls. In subgroup analyses, previous cesarean section was less common in extreme to very PTBs (17.6%) compared with moderate to late PTBs (26.5%) (p=0.036).

**Table 3:** Obstetric and gynecological history of women included in a matched case–control study of preterm birth, overall and according to prematurity status and degree of prematurity, in a tertiary referral hospital, Cyprus, 2019–2022 (N=978)

*Characteristics*	*Overall* *(N=978)* *n (%)*	*Prematurity status (N=978)*			*Degree of prematurity (N=489)*		
		*Preterm* *birth* *(<37 weeks)* *(N=489)* *n (%)*	*Term birth* *(≥37 weeks)* *(N=489)* *n (%)*	*p*	*Extreme* *to very* *preterm* *(<32 weeks)* *(N=142)* *n (%)*	*Moderate to* *late preterm* *(32 to <37* *weeks)* *(N=347)* *n (%)*	*p*
Parity* (live births) (N=977)							
Nulliparous (0)	457 (46.8)	241 (49.3)	216 (44.3)	0.124^a^	70 (49.3)	171 (49.3)	0.987^b^
Primiparous (1)	324 (33.2)	155 (31.7)	169 (34.6)		44 (31.0)	111 (32.0)	
Multiparous (2–4)	187 (19.1)	89 (18.2)	98 (20.1)		27 (19.0)	62 (17.9)	
Grand multiparous (≥5)	9 (0.9)	4 (0.8)	5 (1.0)		1 (0.7)	3 (0.9)	
Gynecological surgeries**							
Yes	418 (42.7)	234 (47.9)	184 (37.6)	**0.001^c^**	65 (45.8)	169 (48.7)	0.556^b^
No	560 (57.3)	255 (52.1)	305 (62.4)		77 (54.2)	178 (51.3)	
**Previous cesarean section**							
Yes	208 (21.3)	117 (23.9)	91 (18.6)	**0.045^c^**	25 (17.6)	92 (26.5)	**0.036^b^**
No	770 (78.7)	372 (76.1)	398 (81.4)		117 (82.4)	255 (73.5)	
**Previous abortion**							
Yes	63 (6.4)	27 (5.5)	36 (7.4)	0.358^c^	10 (7.0)	17 (4.9)	0.346^b^
No	915 (93.6)	462 (94.5)	454 (92.6)		132(93.0)	330 (95.1)	
**Previous miscarriage**							
Yes	197 (20.1)	113 (23.1)	84 (17.2)	0.054^c^	34 (23.9)	79 (22.8)	0.779^b^
No	781 (79.9)	376 (76.9)	405 (82.8)		108 (76.1)	268 (77.2)	
**Breast feeding in previous births** (N=468)							
Yes	362 (77.4)	162 (75.7)	200 (78.7)	0.770^c^	47 (74.6)	115 (76.2)	0.809^b^
No	106 (22.6)	52 (24.3)	54 (21.3)		16 (25.4)	36 (23.8)	
**History of preterm birth** (N=512)							
Yes	92 (18.0)	61 (24.5)	32 (11.8)	0.074^c^	18 (24.7)	43 (24.4)	0.970^b^
No	420 (82.0)	188 (75.5)	232 (88.2)		55 (75.3)	133 (75.6)	

IQR: Interquartile Range. Notes: Percentages are calculated within each category of the corresponding variable. Bold values indicate statistical significance.

a Differences between cases (preterm birth) and controls (term birth) were analyzed using conditional logistic regression.

b Differences between categories of preterm birth (extreme to very preterm vs moderate to late preterm) were analyzed using chi-squared test.

c Differences between cases (preterm birth) and controls (term birth) were analyzed using McNemar’s test for paired nominal data.

* Number of pregnancies that resulted in birth. Parity categories: Nulliparous refers to women with no previous live births; Primiparous refers to women with one previous live birth; Multiparous refers to women with two to four previous live births; Grand multiparous refers to women with five or more previous live births.

** Gynecological surgeries include previous cesarean section, abortion procedures, and dilation and curettage (D&C).

### Factors associated with preterm birth (preterm vs term)

Univariable logistic regression analysis (Table 4) identified that maternal age was associated with PTB, with each additional year associated with higher odds of PTB (OR=1.20; 95% CI: 1.06–1.36). Among obstetric history variables, previous gynecological surgery (OR=1.55; 95% CI: 1.19–2.02), previous cesarean section (OR=1.40; 95% CI: 1.02–1.93), and miscarriage (OR=1.44; 95% CI: 1.05–1.97) were significantly associated with increased odds of PTB. In contrast, maternal education level, occupation, BMI, smoking, drug or alcohol use, abortion, and breastfeeding were not significantly associated with PTB.

**Table 4:** Univariable conditional and binary logistic regression analyses examining associations between sociodemographic characteristics, health behaviors, obstetric history and preterm birth status among women participating in a matched case–control study, in a tertiary referral hospital, Cyprus, 2019–2022 (N=978)

*Variables*	*Prematurity status* *[preterm birth (n=489) vs term birth* *(n=489)]* *(N=978)*		*Degree of prematurity* *[extreme to very (n=142) vs moderate* *to late (n=347)] (N=489)*	
	*OR (95% CI)*	*p*	*OR (95% CI)*	*p*
**Sociodemographic**				
**Maternal age** (years)	1.20 (1.06–1.36)	**0.005**	1.03 (0.99–1.06)	0.131
**Maternal age categories** (years)				
<20 vs 20–34	1.17 (0.39–3.47)	0.781	0.73 (0.20–2.08)	0.583
≥35 vs 20–34	0.71 (0.34–1.48)	0.356	1.27 (0.79–2.03)	0.313
**Maternal country**				
Cyprus vs Other	-	-	0.68 (0.46–1.01)	0.055
**Maternal education** (N=949)				
Primary vs Tertiary	1.30 (0.65–2.61)	0.457	0.49 (0.20–1.25)	0.135
Secondary vs Tertiary	0.96 (0.70–1.32)	0.807	0.64 (0.41–0.99)	**0.043**
**Maternal occupation** (N=944)				
Unemployed vs Employed	0.85 (0.58–1.26)	0.424	0.53 (0.29–0.97)	**0.041**
Housewife vs Employed	0.96 (0.63–1.46)	0.836	1.01 (0.60–1.71)	0.958
Asylum seeker vs Employed	1.17 (0.56–2.47)	0.681	0.19 (0.07–0.53)	**0.002**
**Marital status** (N=965)				
Engaged/in a relationship vs Married	0.80 (0.55–1.16)	0.231	0.90 (0.53–1.55)	0.714
Single/divorced vs Married	0.78 (0.51–1.19)	0.257	0.45 (0.23–0.87)	**0.017**
**Paternal country**				
Cyprus vs Other	0.95 (0.62–1.47)	0.825	0.65 (0.44–0.91)	**0.034**
**Health behaviors**				
**Maternal measurements**				
Weight	1.00 (0.99–1.00)	0.822	0.99 (0.98–1.01)	0.319
Height	1.00 (0.98–1.01)	0.734	1.02 (0.99–1.05)	0.231
BMI	1.00 (0.99–1.00)	0.309	0.97 (0.93–1.01)	0.095
**Smoking**				
Yes vs No	0.89 (0.63–1.25)	0.487	0.99 (0.59–1.67)	0.978
**Drugs of abuse/alcohol consumption**				
Yes vs No	2.00 (0.37–10.92)	0.423	0.81 (0.08–7.89)	0.858
**Obstetric and gynecological history**				
**Parity** (N=977)				
Primiparous vs Nulliparous	0.80 (0.59–1.09)	0.801	0.97 (0.620–1.513)	0.888
Multiparous vs Nulliparous	0.78 (0.54–1.13)	0.783	1.06 (0.63–1.81)	0.819
Great multiparous vs Nulliparous	0.66 (0.17–2.54)	0.663	0.81 (0.08–1.19)	0.686
**Gynecological surgeries**				
Yes vs No	1.55 (1.19–2.02)	**0.001**	0.70 (0.63–1.37)	0.697
**Previous C-section**				
Yes vs No	1.40 (1.02–1.93)	**0.038**	0.63 (0.39–1.03)	0.064
**Previous abortion**				
Yes vs No	0.74 (0.44–1.23)	0.243	1.47 (0.66–3.30)	0.349
**Previous miscarriage**				
Yes vs No	1.44 (1.05–1.97)	**0.023**	1.07 (0.67–1.69)	0.779
**Breast feeding in previous births** (N=468)				
Yes vs No	0.88 (0.50–1.56)	0.662	0.95 (0.48–1.87)	0.876
**History of preterm birth** (N=512)				
Yes vs No	1.81 (0.98–3.34)	0.056	1.09 (0.59–2.05)	0.779

OR: odds ratio. CI: confidence interval. BMI: body mass index. Reference category for comparison of prematurity status is term birth (gestational week at delivery ≥37 weeks). Reference category for comparison of degree of prematurity is moderate to late (32 to <37 weeks). For maternal education, the reference was tertiary education; for maternal occupation, employed; for marital status, married; and for maternal and paternal country of origin, Cyprus. Parity was referenced to nulliparous women. For smoking, alcohol consumption, drug abuse, previous C-section, previous miscarriage, previous preterm birth, and history of gynecological surgeries, the reference category was ‘no’. Maternal age and BMI were treated as continuous variables. Conditional logistic regression was used for the comparison of prematurity status (preterm births vs term birth), and binary logistic regression for the comparison of degree of prematurity (extreme to very preterm vs moderate to late preterm).

In the multivariable model (Table 5), the association between maternal age and PTB remained significant (AOR=1.21; 95% CI: 1.06–1.38). However, when maternal age was analyzed categorically (<20, 20–34, and ≥35 years), no independent associations with PTB were identified after adjustment. The strength of associations with obstetric history variables increased after adjustment, with women having a history of gynecological surgery showing 74.3% higher odds of PTB (AOR=1.74; 95% CI: 1.30–2.34), and those with a previous cesarean section showing 87.0% higher odds (AOR=1.87; 95% CI: 1.28–2.73). Previous miscarriage was also associated with prematurity (AOR=1.44; 95% CI: 1.03–2.00). Primiparity was associated with a reduced risk of PTB compared to nulliparity (AOR=0.71; 95% CI: 0.52–0.98).

**Table 5:** Multivariable conditional and binary logistic regression analyses of factors associated with preterm birth status and degree of prematurity among women included in a matched case–control study, in a tertiary referral hospital, Cyprus, 2019–2022 (N=978)

*Variables*	*Prematurity status* *[preterm birth (n=489) vs term* *birth (n=489)]* *(N=978)*		*Degree of prematurity* *[extreme to very (n=142) vs* *moderate to late (n=347)]* *(N=489)*	
	*AOR (95% CI)*	*p*	*AOR (95% CI)*	*p*
**Sociodemographic**				
**Maternal age** (years)	1.21 (1.06–1.38)	**0.005**	1.03 (0.986–1.069)	0.197
**Maternal age categories** (years)				
<20 vs 20–34	1.16 (0.39–3.50)	0.787	0.99 (0.08–0.60)	**0.004**
≥35 vs 20–34	0.69 (0.33–1.45)	0.331	1.22 (0.27–3.01)	0.989
**Maternal country**				
Cyprus vs Other	-	-	0.76 (0.48–1.21)	0.243
**Maternal education** (N=949)				
Primary vs Tertiary	1.39 (0.67–2.86)	0.375	0.62 (0.22–1.78)	0.376
Secondary vs Tertiary	1.02 (0.73–1.42)	0.903	0.75 (0.46–1.22)	0.245
**Maternal occupation** (N=944)				
Unemployed vs Employed	0.84 (0.55–1.28)	0.410	0.63 (0.32–1.24)	0.183
Housewife vs Employed	1.01 (0.64–1.61)	0.965	1.14 (0.61–2.13)	0.684
Asylum seeker vs Employed	1.00 (0.46–2.20)	1.000	0.19 (0.05–0.67)	**0.010**
**Marital status** (N=965)				
Engaged/in a relationship vs Married	0.80 (0.55–1.16)	0.231	1.00 (0.57–1.77)	0.995
Single/divorced vs Married	0.78 (0.51–1.19)	0.257	0.52 (0.25–1.08)	0.078
**Paternal country**				
Cyprus vs Other	0.89 (0.57–1.41)	0.632	0.82 (0.43–1.59)	0.559
**Health behaviors**				
**Maternal measurements**				
Weight	1.00 (0.99–1.02)	0.447	1.02 (0.98–1.07)	0.248
Height	0.98 (0.96–1.00)	0.120	1.01 (0.98–1.05)	0.446
BMI	0.99 (0.99–1.01)	0.384	0.96 (0.92–0.99)	**0.036**
**Smoking**				
Yes vs No	0.90 (0.63–1.27)	0.539	0.98 (0.57–1.67)	0.931
**Drugs of abuse/alcohol consumption**				
Yes vs No	2.06 (0.37–11.58)	0.414	0.84 (0.08–8.67)	0.886
**Obstetric and gynecological history**				
**Parity** (N=977)				
Primiparous vs Nulliparous	0.71 (0.52–0.98)	**0.039**	0.89 (0.56–1.43)	0.631
Multiparous vs Nulliparous	0.71 (0.48–1.04)	0.081	1.11 (0.60–2.04)	0.749
Great multiparous vs Nulliparous	0.72 (0.17–3.02)	0.663	1.03 (0.10–11.17)	0.978
**Gynecological surgeries**				
Yes vs No	1.74 (1.30–2.34)	**<0.001**	0.85 (0.55–1.32)	0.466
**Previous C-section**				
Yes vs No	1.87 (1.28–2.73)	**0.001**	0.54 (0.30–0.99)	**0.044**
**Previous abortion**				
Yes vs No	0.75 (0.44–1.30)	0.310	1.36 (0.56–3.32)	0.500
**Previous miscarriage**				
Yes vs No	1.44 (1.034–2.00)	**0.031**	0.94 (0.57–1.54)	0.798
**Breast feeding in previous births** (N=468)				
Yes vs No	0.88 (0.44–1.73)	0.705	0.72 (0.34–1.53)	0.392
**History of preterm birth** (N=512)				
Yes vs No	1.51 (0.98–3.36)	0.235	1.25 (0.63–2.45)	0.522

AOR: adjusted odds ratio. CI: confidence interval. BMI: body mass index. All models were adjusted for maternal age, country, education level, BMI, smoking, and parity. Reference category for comparison of prematurity status is term birth (gestational week at delivery ≥37 weeks). Reference category for comparison of degree of prematurity is moderate to late (32 to <37 weeks). For maternal education, the reference was tertiary education; for maternal occupation, employed; for marital status, married; and for maternal and paternal country of origin, Cyprus. Parity was referenced to nulliparous women. For smoking, alcohol consumption, drug abuse, previous C-section, previous miscarriage, previous preterm birth, and history of gynecological surgeries, the reference category was ‘no’. Maternal age and BMI were treated as continuous variables. Conditional logistic regression was used for the comparison of prematurity status (preterm birth vs term birth), and binary logistic regression for the comparison of degree of prematurity (extreme to very preterm vs moderate to late preterm).

### Factors associated with the degree of prematurity (extreme to very vs moderate to late)

In the univariable analysis (Table 4), several sociodemographic factors were associated with the risk of extreme to very preterm delivery. Women with secondary education had lower odds of extreme prematurity compared with those with tertiary education (OR=0.64; 95% CI: 0.41–0.99). Unemployment was also associated with reduced odds compared with employment (OR=0.53; 95% CI: 0.29–0.97). Asylum seeker status also showed an association compared with those not seeking asylum (OR=0.19; 95% CI: 0.07–0.54). Being single or divorced was associated with lower odds of extreme preterm delivery compared with being married (OR=0.45; 95% CI: 0.23–0.87). In addition, paternal non-Cypriot origin was associated with reduced odds of extreme preterm birth (OR=0.65; 95% CI: 0.44–0.91).

After adjustment for covariates of interest (Table 5), most of these associations lost statistical significance. In the subgroup analysis, women aged <20 years demonstrated significantly lower odds of extreme to very PTB compared with moderate to late PTB, whereas no significant association was observed among women aged ≥35 years, compared with the reference group (age 20–34 years). The sociodemographic factor that remained significant was asylum seeker status, with asylum seekers continuing to show markedly lower odds of extreme to very preterm delivery (AOR=0.19; 95% CI: 0.05–0.67). Regarding health behaviors, maternal BMI emerged as a significant factor, with higher BMI associated with lower odds of extreme prematurity (AOR=0.96; 95% CI: 0.92–0.99). In addition, previous cesarean section, while not significant in univariable analysis, was associated with reduced odds of extreme prematurity after adjustment (AOR=0.54; 95% CI: 0.30–0.99).

The conditional logistic regression model accounting for the matched study design showed adequate fit, with a log-likelihood of -58.0 and an AIC=150.1. VIF values ranged from 1.08 to 2.21, with all values below the predefined threshold of 2.5, indicating no problematic multicollinearity. Leave-one-stratum-out diagnostics showed no substantial changes in the direction or magnitude of the estimated associations after sequential exclusion of individual matched strata. Interaction analysis using binary logistic regression did not identify any statistically significant interaction effects between the variables examined. For the binary logistic regression model comparing extreme very PTB with moderate to late PTB, calibration was good, as indicated by a non-significant Hosmer–Lemeshow test (χ²=13.38, p=0.10). The model likelihood was -58.0 with an AIC=150.1. Cook’s distance analysis did not reveal any observations with undue influence, and VIF values were within acceptable limits.

## DISCUSSION

This matched case-control study investigated a wide range of sociodemographic, behavioral, and obstetric history factors associated with PTB, as well as with its severity. This study indicated that advanced maternal age, a history of gynecological surgeries, miscarriage, and previous cesarean section were associated with an increased probability of PTB. In contrast, parity, higher maternal BMI, and asylum-seeking status were identified as factors for lower odds of prematurity. Cesarean section was also found to be associated with a lower likelihood of extreme to very preterm delivery, highlighting the complexity of its role in PTB risk.

Although maternal age categories were not independently associated with PTB after adjustment, increasing maternal age overall remained associated with higher PTB risk, highlighting the complex relationship between maternal age and adverse pregnancy outcomes^10^. Older maternal age has been associated with biological and clinical changes that may partly explain the observed association with PTB, including higher rates of cervical insufficiency and reduced endometrial receptivity, which may influence implantation and pregnancy maintenance^11^. In addition, chronic conditions such as hypertensive disorders, gestational diabetes, thyroid dysfunction, and cardiovascular disease are more prevalent in this group and may also be associated with pregnancy complications and adverse outcomes^12-14^. In our analysis, advanced maternal age remained associated with PTB even after adjustment for other factors, underscoring its significance as a determinant of perinatal outcomes. These findings may help inform antenatal risk assessment and support further research exploring the relationship between delayed childbearing and maternal and child health outcomes.

The finding that a history of gynecological surgery increased the probability of PTB is consistent with previous evidence. Although this study did not differentiate between types of procedures, earlier research has shown that cervical and uterine interventions are associated with an elevated risk of adverse pregnancy outcomes^15^. These procedures may cause cervical insufficiency or uterine scarring, which may affect the structural integrity of the reproductive tract and may interfere with implantation, placental development, and cervical competence^16^.

Similarly, the association between previous cesarean section and increased risk of PTB is well documented^17^. Prior cesarean delivery may be associated with abnormal placentation in subsequent pregnancies, including placenta previa and placenta accreta, when the uterine scar disrupts the endometrial–myometrial interface and impairs normal trophoblast invasion^18^. Such placental abnormalities may be associated with both spontaneous and medically indicated PTB, often requiring early delivery to prevent maternal or fetal compromise^19^. Uterine scarring may also increase the risk of rupture or dehiscence, particularly during a trial of labor after cesarean, which may influence clinical decision-making regarding delivery timing^20^. These findings should be interpreted cautiously and warrant further investigation in prospective studies.

A history of miscarriage was also associated with PTB, consistent with previous evidence^21^. Recurrent pregnancy loss may reflect underlying uterine anomalies, hormonal or thyroid dysfunction, or chronic endometrial inflammation, all of which are associated with adverse pregnancy outcomes^22^. In addition to these biological mechanisms, the psychological burden of miscarriage has been shown to influence neuroendocrine pathways, such as the hypothalamic–pituitary–adrenal axis, which may be associated with increased risk of spontaneous PTB^23^.

The effect of parity against PTB observed in our study is in line with previous reports^4,24,25^. Beyond biological adaptation, multiparous women may benefit from prior pregnancy experience and greater awareness of warning signs. Evidence suggests that nulliparous women are more likely to initiate ANC late and to attend fewer visits, factors associated with increased risk of adverse outcomes, including PTB^26^.

This study also explored the severity of PTB, distinguishing between extreme to very and moderate to late preterm births. Asylum seeker status was associated with a lower likelihood of extreme PTB in our study. Although migrant populations are often considered at higher risk for adverse perinatal outcomes due to socioeconomic disadvantage, language barriers, and limited access to healthcare^27^, the ‘healthy migrant effect’ has been proposed as a possible explanation for unexpectedly favorable outcomes among certain groups^28^. This phenomenon refers to the positive self-selection of migrants, who are often younger, healthier, and more resilient than both the population in their country of origin and the host population^29^. Healthier behaviors, stronger social cohesion, and supportive cultural practices around pregnancy may further contribute to these outcomes^29^. However, the strength of the ‘healthy migrant effect’ varies across settings and may diminish over time with acculturation, particularly where structural barriers limit healthcare access. Thus, while the association observed among asylum seekers in Cyprus may reflect this phenomenon, it should be interpreted with caution and in the context of local health and social support systems.

Higher maternal BMI was also found to be associated with extreme PTB, in line with previous population-based studies^30^. A possible explanation is that greater adiposity may be associated with metabolic or hormonal stability linked to longer gestation^31^. However, this apparent effect must be interpreted with caution, as elevated BMI is also a well-established risk factor for gestational diabetes, hypertensive disorders, and other obstetric complications^32^. The complex relationship between maternal BMI and PTB highlights the need for further research, while reinforcing the importance of balanced preconception counselling and tailored antenatal care to optimize outcomes across the BMI spectrum.

Interestingly, while previous cesarean section was identified to be associated with PTB overall, it was associated with a reduced likelihood of extreme PTB in the subgroup analysis. This paradox may partly reflect closer prenatal monitoring and more intensive clinical management of women with prior Cesarean section, which might help delay delivery to later gestational ages^33^. In addition, the scheduling of elective cesarean deliveries in the late preterm period (34–36 weeks) may shift births away from more extreme gestations. Obstetricians may also opt for earlier delivery to reduce risks of uterine rupture or abnormal placentation, thereby preventing emergencies at very early stages^20^. While this approach could increase late PTB, it may simultaneously reduce extreme PTB. However, the literature on this association is inconsistent, with variations across populations and clinical practices^34^. Further research is warranted to clarify the moderating role of prior cesarean section on gestational age distribution and to distinguish between spontaneous and medically indicated PTB.

### Limitations

Several limitations of the study should be noted. The retrospective observational design precludes causal inference and is subject to selection and information bias, while incomplete or variable-quality medical records may have led to misclassification despite standardized extraction procedures. Residual confounding from unmeasured factors, including antenatal care, psychological status, or nutrition, cannot be excluded. The single-center setting limits generalizability, and some self-reported variables may be affected by recall bias, particularly among non-native language speakers. Additionally, both spontaneous and medically indicated PTBs were included, as the retrospective medical records did not consistently allow reliable differentiation between these subtypes, which may have influenced comparisons with studies examining spontaneous PTB exclusively. Some subgroup analyses included relatively small numbers of participants within specific maternal age categories, particularly among women aged <20 years, which may have affected the precision and stability of the estimates. Moreover, BMI calculations were based on weight recorded at the first prenatal visit, which may not fully reflect true pre-pregnancy weight and could be subject to reporting inaccuracies. Behavioral variables such as smoking and drug/alcohol use were based on recorded information referring to the period before and/or during pregnancy, and may not have fully captured changes in behaviors across gestation. Data related to access and adequacy of prenatal care were unavailable and could therefore not be evaluated in the present study. Although the study period overlapped with the COVID-19 pandemic, adjustment or stratified analyses according to year or pandemic period were not feasible because anonymization procedures following data extraction prevented retrieval of individual birth years in the final analytical dataset. Consequently, potential temporal effects related to the pandemic on PTB rates and associated risk factors cannot be excluded.

Despite these limitations, this study identifies key associated factors for PTB in Cyprus with important clinical and public health implications. Covariates such as advanced maternal age, gynecological surgery, prior Cesarean section, and miscarriage highlight the need for early risk identification and tailored monitoring of high-risk pregnancies. Culturally sensitive health education may further support engagement with prenatal care, particularly among vulnerable or migrant populations. From a public health perspective, optimizing the continuity and quality of antenatal care remains essential, while unexpected associations, including higher BMI and asylum-seeker status, warrant cautious interpretation and further context-specific research.

### Future research

The findings align with existing literature and reinforce the multifactorial nature of PTB. Future research should prioritize large longitudinal cohorts to clarify the roles of BMI, asylum-seeker status, and previous Cesarean section in relation to PTB timing and severity, and to distinguish between spontaneous and medically indicated cases. Qualitative studies could further elucidate healthcare barriers among high-risk groups. In Cyprus, additional research on maternal medical history, mental health, and lifestyle behaviors is needed to inform targeted, culturally sensitive interventions to reduce the burden of PTB.

## CONCLUSIONS

This matched case–control study provides new evidence on the multifactorial nature of PTB in Cyprus. Independent covariates included advanced maternal age, history of gynecological surgery, miscarriage, and previous Cesarean section, while parity, higher maternal BMI, and asylum-seeking status were associated with reduced risk. The findings suggest a potentially complex relationship between Cesarean delivery, maternal BMI, and PTB severity, warranting further investigation in larger prospective studies. Improved understanding of these associations may help support future approaches to antenatal risk assessment and management among higher risk pregnancies. Given Cyprus’s persistently high PTB rates, integrated care pathways and targeted public health initiatives are urgently needed to improve maternal and neonatal outcomes.

## Supplementary Material



## Data Availability

The data supporting this research are available from the authors on reasonable request.

## References

[1] World Health Organization. Born too soon: decade of action on preterm birth; May 9, 2023. Accessed June 11, 2026. https://www.who.int/publications/i/item/9789240073890

[2] Ohuma EO, Moller AB, Bradley E, et al. National, regional, and global estimates of preterm birth in 2020, with trends from 2010: a systematic analysis. Lancet. 2023;402(10409):1261-1271. doi:10.1016/S0140-6736(23)00878-4 37805217

[3] Kiserud T, Piaggio G, Carroli G, Widmer M, Carvalho J, Neerup Jensen L, et al. The World Health Organization fetal growth charts: a multinational longitudinal study of ultrasound biometric measurements and estimated fetal weight. PLoS Med. 2017;14(1):e1002220. doi:10.1371/journal.pmed.1002220 28118360 PMC5261648

[4] Goldenberg RL, Culhane JF, Iams JD, Romero R. Epidemiology and causes of preterm birth. Lancet. 2008;371(9606):75-84. doi:10.1016/S0140-6736(08)60074-4 18177778 PMC7134569

[5] Walani SR. Global burden of preterm birth. Int J Gynaecol Obstet. 2020;150(1):31-33. doi:10.1002/ijgo.13195 32524596

[6] Ministry of Health, Republic of Cyprus, Health Monitoring Unit. Important perinatal indicators Cyprus 2015–2023; February, 2025. Accessed June 11, 2026. https://www.gov.cy/media/sites/24/2024/05/Important-Perinatal-Indicators-Cyprus_2015-2023.pdf

[7] Stylianou-Riga P, Kouis P, Kinni P, et al. Maternal socioeconomic factors and the risk of premature birth and low birth weight in Cyprus: a case-control study. Reprod Health. 2018;15(1):157. doi:10.1186/s12978-018-0603-7 30231873 PMC6146509

[8] Di Renzo GC, Bartha JL, David AL, et al. Predicting and preventing preterm birth in Europe: current challenges and gaps. J Matern Fetal Neonatal Med. 2025;38(1):2547687. doi:10.1080/14767058.2025.2547687 40841308

[9] von Elm E, Altman DG, Egger M, et al. The Strengthening the Reporting of Observational Studies in Epidemiology (STROBE) statement: guidelines for reporting observational studies. J Clin Epidemiol. 2008;61(4):344-349. doi:10.1016/j.jclinepi.2007.11.008 18313558

[10] Kenny LC, Lavender T, McNamee R, O’Neill SM, Mills T, Khashan AS. Advanced maternal age and adverse pregnancy outcome: evidence from a large contemporary cohort. PLoS One. 2013;8(2):e56583. doi:10.1371/journal.pone.0056583 23437176 PMC3577849

[11] Pathare ADS, Loid M, Saare M, et al. Endometrial receptivity in women of advanced age: an underrated factor in infertility. Hum Reprod Update. 2023;29(6):773-793. doi:10.1093/humupd/dmad019 37468438 PMC10628506

[12] Kim C. Maternal outcomes and follow-up after gestational diabetes mellitus. Diabet Med. 2014;31(3):292-301. doi:10.1111/dme.12382 24341443 PMC3944879

[13] Casey BM, Thom EA, Peaceman AM, et al. Treatment of subclinical hypothyroidism or hypothyroxinemia in pregnancy. N Engl J Med. 2017;376(9):815-825. doi:10.1056/NEJMoa1606205 28249134 PMC5605129

[14] Kim MJ. Pregnancy at late maternal age and future cardiovascular health. Cardiovasc Prev Pharmacother. 2019;1(2):50-56. doi:10.36011/cpp.2019.1.e9

[15] Kim YR, Na ED, Jung JE, Moon JH, Lee JY. Clinical features at the time of non-hysteroscopic myomectomy before pregnancy, which affect adverse pregnancy outcomes: a retrospective cohort study. BMC Pregnancy Childbirth. 2022;22(1):896. doi:10.1186/s12884-022-05240-7 36463110 PMC9719619

[16] Hooker AB, de Leeuw RA, Emanuel MH, Mijatovic V, Brolmann HAM, Huirne JAF. The link between intrauterine adhesions and impaired reproductive performance: a systematic review of the literature. BMC Pregnancy Childbirth. 2022;22(1):837. doi:10.1186/s12884-022-05164-2 36376829 PMC9664654

[17] Reddy UM. Unravelling caesarean delivery as a risk factor for antepartum stillbirth. BJOG. 2015;122(11):1475. doi:10.1111/1471-0528.13531 26234883

[18] Jauniaux E, Bhide A. Prenatal ultrasound diagnosis and outcome of placenta previa accreta after cesarean delivery: a systematic review and meta-analysis. Am J Obstet Gynecol. 2017;217(1):27-36. doi:10.1016/j.ajog.2017.02.050 28268196

[19] Vahanian SA, Lavery JA, Ananth CV, Vintzileos A. Placental implantation abnormalities and risk of preterm delivery: a systematic review and metaanalysis. Am J Obstet Gynecol. 2015;213(4 Suppl):S78-S90. doi:10.1016/j.ajog.2015.05.058 26428506

[20] Stamilio DM, DeFranco E, Paré E, et al. Short interpregnancy interval: risk of uterine rupture and complications of vaginal birth after cesarean delivery. Obstet Gynecol. 2007;110(5):1075-1082. doi:10.1097/01.AOG.0000286759.49895.46 17978122

[21] Bhattacharya S, McCall SJ, Woolner AMF. Recurrent pregnancy loss and subsequent preterm birth: association or causation?. Fertil Steril. 2022;117(4):820-821. doi:10.1016/j.fertnstert.2022.01.038 35367017

[22] Turesheva A, Aimagambetova G, Ukybassova T, et al. Recurrent pregnancy loss etiology, risk factors, diagnosis, and management. Fresh look into a full box. J Clin Med. 2023;12(12):4074. doi:10.3390/jcm12124074 37373766 PMC10298962

[23] Khalesi ZB, Bokaie M. The association between pregnancy-specific anxiety and preterm birth: a cohort study. Afr Health Sci. 2018;18(3):569-575. doi:10.4314/ahs.v18i3.14 30602989 PMC6306999

[24] Shah PS, Balkhair T, Ohlsson A, Beyene J, Scott F, Frick C. Intention to become pregnant and low birth weight and preterm birth: a systematic review. Matern Child Health J. 2011;15(2):205-216. doi:10.1007/s10995-009-0546-2 20012348

[25] Koullali B, van Zijl MD, Kazemier BM, et al. The association between parity and spontaneous preterm birth: a population based study. BMC Pregnancy Childbirth. 2020;20(1):233. doi:10.1186/s12884-020-02940-w 32316915 PMC7175552

[26] World Health Organization. WHO recommendations on antenatal care for a positive pregnancy experience; 2016. Accessed June 11, 2026. https://iris.who.int/server/api/core/bitstreams/9dccde13-3593-4a22-9237-61abe5a3c6b7/content 28079998

[27] Heslehurst N, Brown H, Pemu A, Coleman H, Rankin J. Perinatal health outcomes and care among asylum seekers and refugees: a systematic review of systematic reviews. BMC Med. 2018;16(1):89. doi:10.1186/s12916-018-1064-0 29890984 PMC5996508

[28] Miller LS, Robinson JA, Cibula DA. Healthy immigrant effect: preterm births among immigrants and refugees in Syracuse, NY. Matern Child Health J. 2016;20(2):484-493. doi:10.1007/s10995-015-1846-3 26525555

[29] Stanek M, Juárez SP, Requena M. Challenges in current research on immigrant health: insights from Spain. In: Stanek M, Juárez SP, Requena M, eds. Multidisciplinary perspectives on immigrant health: new insights from Spain. Springer; 2025:1-20. IMISCOE Research Series. doi:10.1007/978-3-031-82352-7_1

[30] Ehrenberg HM, Iams JD, Goldenberg RL, et al. Maternal obesity, uterine activity, and the risk of spontaneous preterm birth. Obstet Gynecol. 2009;113(1):48-52. doi:10.1097/AOG.0b013e318191c818 19104359 PMC2790406

[31] Kabbani N, Blüher M, Stepan H, et al. Adipokines in pregnancy: a systematic review of clinical data. Biomedicines. 2023;11(5):1419. doi:10.3390/biomedicines11051419 37239090 PMC10216846

[32] Lourenço J, Guedes-Martins L. Pathophysiology of maternal obesity and hypertension in pregnancy. J Cardiovasc Dev Dis. 2025;12(3):91. doi:10.3390/jcdd12030091 40137089 PMC11942925

[33] Thanh BYL, Lumbiganon P, Pattanittum P, et al. Mode of delivery and pregnancy outcomes in preterm birth: a secondary analysis of the WHO global and multi-country surveys. Sci Rep. 2019;9(1):15556. doi:10.1038/s41598-019-52015-w 31664121 PMC6820722

[34] Behrman RE, Butler AS, Institute of Medicine (US) Committee on Understanding Premature Birth and Assuring Healthy Outcomes, eds. Preterm birth: causes, consequences, and prevention. Washington (DC): National Academies Press (US); 2007. doi:10.17226/11622 20669423

