# Supplementary material for: Sociodemographic, behavioral, and obstetric factors associated with preterm birth and its severity: A matched case-control study in Cyprus
Source: Eur J Midwifery. 2026 Jul 24;10:10.18332/ejm/224190. doi: 10.18332/ejm/224190 (PMC13401238; doi:10.18332/ejm/224190)
Supplement: Supplementary file 1 [file EJM-10-33-s1.pdf]

STROBE statement - Completed checklist for observational studies

Sociodemographic, behavioural, and obstetric predictors of preterm birth and Its severity in Cyprus: a matched case-control study

Section	Item	STROBE recommendation	Reported on page / line	Information reported in manuscript
Title and abstract	1(a)	Indicate the study's design with a commonly used term in the title or the abstract.	Title page and Abstract, p.1, lines 1-13	The title identifies the design as a matched case-control study. The Methods section of the abstract also states that a matched case-control study was conducted.
Title and abstract	1(b)	Provide in the abstract an informative and balanced summary of what was done and what was found.	Abstract, p.1, lines 4-31	The abstract summarises the background, aim, setting/design, participants, statistical approach, principal findings, and conclusion.
Introduction	2	Explain the scientific background and rationale for the investigation being reported.	Introduction, p.2, lines 33-62	The introduction defines PTB, summarises its global burden, describes known determinants, and explains the need for Cyprus-specific evidence.
Introduction	3	State specific objectives, including any prespecified hypotheses.	Introduction, p.2, lines 63-67	The study objective was to investigate sociodemographic, behavioural, and obstetric history factors associated with PTB in Cyprus and to examine whether these factors differed by degree of prematurity.
Methods	4	Present key elements of study design early in the paper.	Methods, section 2.1, p.3, lines 68-80	The manuscript presents the matched case-control design based on hospital medical records at the start of the Methods section.
Methods	5	Describe the setting, locations, and relevant dates, including periods of recruitment, exposure, follow-up, and data collection.	Methods, sections 2.1-2.2, p.3, lines 68-84	The study was conducted at a tertiary referral hospital in Nicosia, Cyprus, under SHSO; eligible women delivered between January 2019 and December 2022.
Methods	6(a)	Case-control study: Give eligibility criteria, and the sources and methods of case ascertainment and control selection. Give the rationale for the choice of cases and controls.	Methods, section 2.2, p.3, lines 81-93	Eligible participants were women giving birth at the tertiary hospital between January 2019 and December 2022. Exclusions were stillbirths, multiple pregnancies, or incomplete medical records. Cases were identified through the hospital annual birth registry and controls were term births selected in a 1:1 ratio.
Methods	6(b)	Case-control study: For matched studies, give matching criteria and the number of controls per case.	Methods, section 2.2, p.3, lines 86-93; Results, p.5, lines 159-161	Controls were matched 1:1 to cases on maternal age (+/-3 years) and country of origin. The analysis included 489 cases and 489 matched controls.
Methods	7	Clearly define all outcomes, exposures, predictors, potential confounders, and effect modifiers. Give diagnostic criteria, if applicable.	Methods, sections 2.4-2.4.1, pp.3-4, lines 98-119	PTB and term birth definitions are provided, PTB severity groups are defined, and potential covariates are grouped into sociodemographic, behavioural, and obstetric/gynecological domains.
Methods	8	For each variable of interest, give sources of data and details of methods of assessment/measurement. Describe comparability of assessment methods if there is more than one group.	Methods, sections 2.2-2.4.1, pp.3-4, lines 85-119	Data were obtained from the hospital annual birth registry and medical records using unique patient identification numbers. Gestational age was taken from medical records, BMI was calculated from first prenatal visit weight and height, and variables were coded in a secure encrypted database.
Methods	9	Describe any efforts to address potential sources of bias.	Methods, sections 2.2 and 2.5, pp.3-5, lines 86-93 and 127-147; Discussion limitations, pp.10-11, lines 328-350	Potential confounding was addressed through matching on maternal age and country of origin, covariate adjustment, SMD assessment, multicollinearity checks, model diagnostics, and influence diagnostics. Limitations discuss potential selection, information, misclassification, residual confounding, and recall bias.
Methods	10	Explain how the study size was arrived at.	Methods/Results, sections 2.2 and 3.1, pp.3 and 5, lines 81-84 and 159-161	The manuscript reports inclusion of eligible women delivering during the 2019-2022 period and the final analysed sample of 978 women (489 cases and 489 controls). A formal sample size or power calculation is not explicitly reported.
Methods	11	Explain how quantitative variables were handled in the analyses. If applicable, describe which groupings were chosen and why.	Methods, sections 2.4.1 and 2.5, pp.4-5, lines 111-123; Tables 1-5	Maternal age, weight, height, and BMI were analysed as continuous variables; parity and maternal age categories were also defined. Continuous variables were summarised as mean (SD) or median (IQR) according to distribution.
Methods	12(a)	Describe all statistical methods, including those used to control for confounding.	Methods, section 2.5, pp.4-5, lines 121-153	The manuscript describes Shapiro-Wilk testing, Wilcoxon signed-rank, McNemar's test, conditional logistic regression, Mann-Whitney U, chi-squared/Fisher's exact tests, binary logistic regression, multivariable models, and adjustment for theoretically relevant covariates.
Methods	12(b)	Describe any methods used to examine subgroups and interactions.	Methods, section 2.5, pp.4-5, lines 130-145; Results, section 3.5, pp.7-8	Subgroup analysis compared extreme to very PTB with moderate to late PTB using unmatched tests and binary logistic regression. Potential interactions were tested using binary logistic regression.

© 2026 Mastrou L. et al. DOI:10.18332/ejm/224190

Section	Item	STROBE recommendation	Reported on page / line	Information reported in manuscript
Methods	12(c)	Explain how missing data were addressed.	Methods, sections 2.3-2.4, pp.3-4, lines 94-101	Records missing essential information across key domains were excluded. Missing data were handled using available-case analysis, and the number of observations for each variable is reported in the relevant tables.
Methods	12(d)	Case-control study: If applicable, explain how matching of cases and controls was addressed.	Methods, sections 2.2 and 2.5, pp.3-5, lines 86-93 and 124-132	Matching criteria are specified as maternal age (+/-3 years) and country of origin with a 1:1 control-to-case ratio. Conditional logistic regression was used for the matched primary comparison.
Methods	12(e)	Describe any sensitivity analyses.	Methods, section 2.5, p.5, lines 140-148; Results, section 3.5, pp.7-8, lines 230-240	Influence and sensitivity diagnostics included Cook's distance for binary logistic models and leave-one-stratum-out diagnostics for conditional logistic models.
Results	13(a)	Report numbers of individuals at each stage of study, including numbers potentially eligible, examined for eligibility, confirmed eligible, included, and analysed.	Results, section 3.1, p.5, lines 159-161; Tables 1-5	The Results report a final analysed sample of 978 women, comprising 489 PTB cases and 489 matched controls. Tables report analysis-specific denominators.
Results	13(b)	Give reasons for non-participation at each stage.	Methods, sections 2.2-2.3, p.3, lines 83-97	The manuscript lists exclusion criteria and states that records missing essential information across key domains were excluded. Detailed numbers excluded at each stage are not explicitly reported in the visible manuscript text.
Results	13(c)	Consider use of a flow diagram.	Not clearly identified in the manuscript file	A flow diagram was not clearly identified in the manuscript text provided. If a participation flow figure exists separately, it should be referenced here.
Results	14(a)	Give characteristics of study participants and information on exposures and potential confounders.	Results, sections 3.1-3.3, pp.5-6, lines 159-190; Tables 1-3, pp.15-19	Participant sociodemographic characteristics, health behaviours, anthropometric measures, and obstetric/gynecological history are reported overall, by PTB status, and by degree of prematurity.
Results	14(b)	Indicate number of participants with missing data for each variable of interest.	Methods, p.4, lines 100-101; Tables 1-5	Available-case analysis is stated and table row labels report variable-specific denominators (e.g., education n=949, occupation n=944, parity n=977).
Results	14(c)	Cohort study: Summarise follow-up time.	Not applicable	Not applicable because this is a matched case-control study rather than a cohort study.
Results	15	Case-control study: Report numbers in each exposure category, or summary measures of exposure.	Results, sections 3.1-3.3, pp.5-6; Tables 1-3, pp.15-19	Numbers/percentages and medians/IQRs are reported separately for cases and controls and for PTB severity groups across exposures and covariates.
Results	16(a)	Give unadjusted estimates and, if applicable, confounder-adjusted estimates and their precision. Make clear which confounders were adjusted for and why they were included.	Results, sections 3.4-3.5, pp.6-8; Tables 4-5, pp.20-23; Methods, p.5, lines 135-139	Univariable and multivariable regression results are reported with OR/aOR, 95% CI, and p-values. The Methods state that models included theoretically relevant variables: maternal age, maternal country of origin, education, parity, smoking, and BMI.
Results	16(b)	Report category boundaries when continuous variables were categorized.	Methods, section 2.4.1, p.4, lines 108-115; Tables 1 and 5	Maternal age categories are reported as <20, 20-34, and >=35 years. Parity categories are reported as nulliparity, primiparity, multiparity, and great multiparity.
Results	16(c)	If relevant, consider translating estimates of relative risk into absolute risk for a meaningful time period.	Not applicable / not reported	The manuscript reports odds ratios for a case-control design. Translation into absolute risk was not reported and is not generally applicable to this matched case-control analysis.
Results	17	Report other analyses done, such as subgroup analyses, interactions, and sensitivity analyses.	Results, section 3.5, pp.7-8, lines 207-240	The manuscript reports subgroup analysis for degree of prematurity, model fit diagnostics, VIF values, leave-one-stratum-out diagnostics, interaction analysis, Hosmer-Lemeshow testing, AIC/log-likelihood, and Cook's distance.
Discussion	18	Summarise key results with reference to study objectives.	Discussion, p.8, lines 242-251	The Discussion summarises the key factors associated with PTB and with degree of prematurity in relation to the study objectives.
Discussion	19	Discuss limitations, taking into account sources of potential bias or imprecision. Discuss both direction and magnitude of potential bias.	Discussion limitations, pp.10-11, lines 328-350	Limitations include retrospective observational design, selection and information bias, misclassification, residual confounding, single-centre setting, recall bias, inability to differentiate spontaneous and medically indicated PTB, small subgroups, BMI measurement limitations, behavioural data limitations, lack of prenatal care data, and possible COVID-19 period effects.
Discussion	20	Give a cautious overall interpretation of results considering objectives, limitations, multiplicity of analyses, similar studies, and other relevant evidence.	Discussion, pp.8-11, lines 242-369	The manuscript interprets findings cautiously, compares them with prior literature, notes inconsistencies, and highlights that prospective studies are needed to confirm associations.
Discussion	21	Discuss the generalisability / external validity of the study results.	Methods setting, p.3, lines 68-80; Discussion limitations, p.10, lines 333-334	The setting is described as a national tertiary referral centre managing most high-risk neonatal cases in Cyprus, while the limitations state that the single-centre setting limits generalisability.
Other information	22	Give the source of funding and the role of the funders.	Declarations, p.12, lines 378-384	The manuscript states that the research received no funding.

© 2026 Mastrou L. et al.
DOI:10.18332/ejm/224190

The content has been provided by the author(s) and has not been reviewed, verified, or endorsed by European Publishing. It may not have undergone peer review. The views, opinions, and recommendations expressed are solely those of the author(s) and do not necessarily reflect the position of European Publishing. European Publishing accepts no responsibility or liability for any consequences arising from the use of, or reliance on, this content.

© 2026 Mastrou L. et al. DOI:10.18332/ejm/224190
